# Significance and agreement between obesity anthropometric measurements and indices in adults: a population-based study from the United Arab Emirates

**DOI:** 10.1186/s12889-021-11650-7

**Published:** 2021-08-31

**Authors:** Ibrahim Mahmoud, Nabil Sulaiman

**Affiliations:** 1 College of Medicine, University of Sharjah, Sharjah, United Arab Emirates; 2 Baker Heart and Diabetes Institute, Melbourne, Victoria Australia

**Keywords:** Agreement, Anthropometric measurements, Cardiometabolic, Obesity, Overweight

## Abstract

**Background:**

The rates of overweight and obese adults in the United Arab Emirates (UAE) have increased dramatically in recent decades. Several anthropometric measurements are used to assess body weight status. Some anthropometric measurements might not be convenient to use in certain communities and settings. The objective of this study was to assess the agreement of four anthropometric measurements and indices of weight status and to investigate their associations with cardiometabolic risks.

**Methods:**

The study design was a cross-section population-based study. Adults living in the Northern Emirates were surveyed. Fasting blood samples, blood pressure readings and anthropometric measurements were also collected.

**Results:**

A total of 3531 subjects were included in this study. The prevalence of obesity/overweight was 66.4% based on body mass index (BMI), 61.7% based on waist circumference (WC), 64.6% based on waist–hip ratio (WHR) and 71% based on neck circumference (NC). There were moderate agreements between BMI and WC and between WC and WHR, with kappa (k) ranging from 0.41 to 0.60. NC showed poor agreement with BMI, WC and WHR, with k ranging from 0 to 0.2. Overweight and obesity based on BMI, WC and WHR were significantly associated with cardiometabolic risks.

**Conclusion:**

Overall, there was a moderate to a poor agreement between BMI, WC, WHR and NC. Particularly, NC showed poor agreement with BMI, WC and WHR. BMI and WC showed better performance for identifying cardiometabolic risks than WHR and NC.

## Introduction

Overweight and obesity pandemics are increasing worldwide. According to the World Health Organisation (WHO), approximately two billion adults worldwide were overweight or obese in 2016, with more than 2.8 million people dying every year due to being overweight or obese [1]. In the United Arab Emirates (UAE), the rates of overweight and obesity are among the highest in the world due to tough weather conditions and rapid urbanisation leading to sedentary lifestyles and consumption of unhealthy fast-food [2].

Anthropometric measurements and indices are quantitative non-invasive tools used to measure the composition of the body. The significance of these measurements and indices is identifying individuals at increased risk of overweight or obesity. A number of epidemiological studies reported a substantial positive association between an increased body weight or obesity and cardiometabolic risks including raised blood cholesterol, high blood pressure and elevated blood glucose [3, 4]. There are several anthropometric measurements and indices, such as body mass index (BMI), waist circumference (WC), waist–hip ratio (WHR) and NC. BMI was developed in the nineteenth century and is the method most commonly used by health professionals worldwide to assess weight status [5]. Nevertheless, BMI is limited in that it does not consider differences in age, sex, bone structure or muscle mass [6]. In the late1990s, the WHO recognised central obesity evaluated by WC or WHR as an important measure for weight status [7]. Moreover, several studies have identified central obesity as a strong predictor of overweight- and obesity-related health problems [8–10]. However, methods to measure central obesity are limited by certain factors, such as lack of ability to differentiate subcutaneous from visceral fat deposition [7]. Recently, NC was identified as a reliable, simple and culturally acceptable measure to assess weight status [11–13]. However, very few population-based studies have attempted to examine whether those anthropometric measurements and indices can be used interchangeably in the clinical and research settings.

This study aimed to assess the agreement of BMI, WC, WHR and NC and whether they can be used interchangeably. An additional objective was to examine the performance of those anthropometric measurements and indices, for identifying cardiometabolic risks in the UAE adult populations.

## Methods

This is a population-based cross-sectional study using secondary data from the UAE National Diabetes and Lifestyle (UAEDIAB) Study. The UAEDIAB Study was a cross-sectional survey designed to investigate the prevalence of diabetes and associated risk factors among Emirati citizens and expatriates. Anthropometric measurements of obesity and blood samples were also collected as part of the UAEDIAB Study.

### Settings

The UAEDIAB Study recruited adults living in the UAE’s Northern Emirates (Sharjah, Ajman, Ras al-Khaimah, Fujairah and Umm al-Quwain).

### Participants

Participants for the UAEDIAB Study were recruited in two phases. In the first phase, adults who lived in the UAE for at least 4 years but were not citizens were approached while applying for their second or subsequent visa renewal. In the second phase, UAE citizens 18 years of age and older were recruited through a household survey following a random selection of regions and were stratified by emirate using a cluster sampling method. In both phases, participants were excluded if they had serious physical disabilities, learning disorders, severe communication barriers or were pregnant. None of the participants was involved in the development of any stage of this study. The methods for the UAEDIAB Study are described in detail elsewhere [14].

### Variables

This study included variables related to participants’ demographic and lifestyle habits (e.g., gender, age, ethnicity, smoking habits and daily physical activity) and anthropometric measurements and indices (BMI, WC, WHR and NC). Systolic and diastolic blood pressures and fasting blood sample assays were used to assess comorbidities including hypertension, diabetes and dyslipidaemia.

### Bias

To standardise data collection procedures, all data collectors attended a comprehensive training workshop that included interview techniques, data collection tools, practical applications and field guidelines. Two data collectors completed each participant’s physical measurements. For waist and hip measurements, each measurement was repeated twice; if the measurements were within 1 cm of one another, the average was calculated, if the difference between the two measurements exceeded 1 cm, the two measurements were repeated. For height, weight and blood pressure each measurement was repeated three times. The average of all three measurements was considered the most accurate and was recorded. Blood pressure was measured at 10-min intervals.

### Data sources and measurements

Blood samples were collected in the morning after overnight fasting, stored in tubes containing the anticoagulant sodium heparin and transported to the reference laboratory. Each participant’s diabetic status was determined using the HbA1c test and the dyslipidaemia status was determined using a lipid profile test. The cutoff values for the tests were defined according to WHO criteria and the American Heart Association guidelines [15, 16].

A trained data collector obtained anthropometric measurements based on the WHO STEPwise Approach to Surveillance (STEPS) protocol [5]. Hip, neck and waist measurements were taken using a non-stretchable plastic tape. WC was measured at the midpoint between the lower margin of the lowest palpable rib and the top of the iliac crest. Hip circumference was measured around the widest portion of the buttocks, with the tape parallel to the floor [5]. NC was measured at the midpoint of the neck’s height, with participants standing upright. Weight and height were measured using a certified SECA stadiometer and weighing scale (Sonashi Mechanical, Dubai, UAE). For all measurements, the subject had to stand still, eyes looking straight ahead with feet close together and arms at the side, and would wear little clothing and no shoes [5].

BMI was calculated by dividing a participant’s weight in kilograms by height in metres squared; it was then defined as normal (18.5–25 kg/m^2^) or overweight/obese (≥ 25 kg/m^2^) and no underweight subjects (< 18.5 kg/m^2^) identified in our study [3]. WC was defined as normal (< 90 cm in males and < 80 cm in females of Asian ethnicity and < 94 cm for males and < 88 cm for females of other ethnicities) or overweight/obese (≥ 90 cm in males and ≥ 80 cm in females of Asian ethnicity and ≥ 102 cm in males and ≥ 88 cm in females of other ethnicities) [5]. WHR was calculated as the waist circumference in centimetres divided by the hip circumference in centimetres; it was defined as normal (< 0.95 in males and <  0.80 in females of Asian ethnicity and <  1.0 in males and <  0.85 in females of other ethnicities) or overweight/obese (≥ 0.95 in males and ≥ 0.80 in females of Asian ethnicity and ≥ 1.0 in males and ≥ 0.85 in females of other ethnicities) [5]. NC was defined as normal (< 35 cm in males and < 32 cm in females) or overweight/obese (≥ 35.5 cm in males and ≥ 32 cm in females).

The interpretation and cutoff values for the agreement between anthropometric measurements and indices were determined based on criteria set by Douglas G. Altman as follow: equal to chance (k = 0.00); poor (0.01 < k ≤ 0.20); fair (0.21 < k ≤ 0.40); moderate (0.41 < k ≤ 0.60); good (0.61 < k ≤ 0.80); excellent (0.81 < k ≤ 0.99); and perfect (k = 1) [17].

### Ethics approval and consent to participate

The Ministry of Health and Prevention Research Ethics Committee approved this study (MOHP/DXB/RE-SUBC/NO-12/2016). All methods were performed in accordance with the Declaration of Helsinki guidelines and regulations. A signed informed consent from all participants was obtained.

### Statistical analysis

No sample size calculation was performed specifically for this study as the study accessed a secondary data from the UAEDIAB Study. Descriptive statistics were used to describe study participants’ characteristics and anthropometric measures by gender. The statistics reported means with standard deviations (SD) for continuous variables and counts with percentages for categorical variables. In bivariate analyses, categorical variables were analysed using a chi-squared test, while continuous variables were analysed using an independent t-test. Concordance or agreement statistical test, Cohen’s kappa (k) test, was conducted to assess the level of agreement among anthropometric measurements and indices based on dichotomous categorical classifications of weight status (normal or overweight/obese). Four binary logistic regression models were conducted using the enter method to assess the association between each anthropometric measurements or indices and diabetes, hypertension and different types of dyslipidaemia. Models were developed using diabetic status (yes/no), hypertension (yes/no) and one of the lipid profiles (yes/no) as dependent variables. Each model was adjusted for one of anthropometric measurements or indices, gender, age, ethnicity, physical activity and smoking status. The statistical significance was set at *p* ≤ 0.05. Statistical Package for Social Science (SPSS), version 26 (IBM Corp, New York) was used to perform the analyses.

The reporting followed the Strengthening the Reporting of Observational Studies in Epidemiology statement for cross-sectional studies.

## Results

A total of 3531 subjects from the UAEDIAB Study were included in this analysis, with 807 (23%) Emirati citizens and 2724 (77%) immigrants. Of the total, 2621 (74.2%) were males, with a mean age of 39 years old (SD: ± 11.3 years) for the entire population. Table 1 shows the stratification of subjects’ characteristics and anthropometric measurements and indices by gender.

**Table 1:** Distribution of participants characteristics by gender, n (%)

Variable	Total	Female	Male	***P*** value
**Age, years**				
18–39	2161 (61.2)	523 (57.5)	1638 (62.5)	
40–59	1199 (34)	322 (35.4)	877 (33.5)	**< 0.0001**
≥ 60	171 (4.8)	65 (7.1)	106 (4)	
**Ethnicity**				
Emirati citizen	807 (22.9)	391 (43)	416 (15.9)	**< 0.0001**
Arab non-Emirati citizen	633 (17.9)	178 (19.6)	455 (17.4)	
Asian non-Arab	1727 (48.9)	251 (27.6)	1476 (56.3)	
Other	364 (10.3)	90 (9.9)	274 (10.5)	
**Smoking status**				
No	2831 (81.1)	828 (94.3)	2003 (76.7)	**< 0.0001**
Yes	658 (18.9)	50 (5.7)	608 (23.3)	
**Physical activity status**				
No	1590 (49.3)	308 (39.7)	1282 (52.4)	**< 0.0001**
Yes	1633 (50.7)	468 (60.3)	1165 (47.6)	
**Body Mass Index (BMI), kg/m**^**2**^				
Normal	1183 (33.6)	278 (30.6)	905 (34.6)	**0.028**
Overweight/obese	2339 (66.4)	630 (69.4)	1709 (65.4)	
**Waist Circumference (WC), cm**				
Normal	1236 (38.3)	247 (29.3)	989 (41.4)	**< 0.0001**
Overweight/obese	1995 (61.7)	595 (70.7)	1400 (58.6)	
**Waist Hip Ratio (WHR), cm**				
Normal	1143 (35.4)	435 (51.7)	708 (29.7)	**< 0.0001**
Overweight/obese	2083 (64.6)	406 (48.3)	1677 (70.3)	
**Neck Circumference (NC), cm**				
Normal	1021 (29)	515 (56.6)	506 (19.3)	**< 0.0001**
Overweight/obese	2508 (71)	395 (43.4)	2113 (80.7)	

*P* values in bold are statistically significant. SD = standard deviation

### Prevalence of overweight/obesity

Table 1 shows overweight/obesity rates were estimated as 66.4% (69.4% for women and 65.4% for men) using BMI, 61.7% (70.7% for women and 58.6% for men) using WC, 64.6% (48.3% for women and 70.3% for men) using WHR, and 71% (43.4% for women and 80.7% for men) using NC. Women had a higher prevalence of overweight/obesity than men based on BMI and WC while men had a higher prevalence based on WHR and NC (*p* ≤ 0.05; Table 1).

### Prevalence of cardiometabolic risk factors

Table 2 shows the prevalence of diabetes, hypertension and dyslipidaemia by gender. Prevalence rates were estimated as 17.3% (18.8% for women and 16.7 for men, *p* = 0.144) for diabetes, 29.5% (18.2% for women and 33.4%, *p* <  0.0001) for hypertension, 44.9% for hypercholesterolemia, 35% for hypertriglyceridemia, 61.7% for low-HDL–C, 44.6% for high-LDL–C, and 78.9% for high cholesterol ratio.

**Table 2:** Prevalence of cardiometabolic risk factors among study participants by gender

Variable	Total, ***N*** = 3531	Female, ***n*** = 910	Male, ***n*** = 2621	***P*** value
**Diabetes Mellitus (DM)**				
No	2916 (82.7)	737 (81.2)	2179 (83.3)	0.144
Yes	608 (17.3)	171 (18.8)	437 (16.7)	
**Hypertension**				
No	2418 (70.5)	712 (81.7)	1706 (66.6)	**< 0.0001**
Yes	1014 (29.5)	160 (18.2)	854 (33.4)	
**Hypercholesterolemia**				
No	1944 (55.1)	533 (58.6)	1411 (53.8)	**0.013**
Yes	1587 (44.9)	377 (41.4)	1210 (46.2)	
**Hypertriglyceridemia**				
No	2296 (65)	711 (78.1)	1585 (60.5)	**< 0.0001**
Yes	1234 (35)	199 (21.9)	1035 (39.5)	
**Low-HDL–C**				
No	1350 (38.3)	399 (34)	951 (36.3)	**< 0.0001**
Yes	2176 (61.7)	507 (56)	1669 (63.7)	
**High-LDL–C**				
No	1956 (55.4)	585 (64.3)	1371 (52.3)	**< 0.0001**
Yes	1575 (44.6)	325 (35.7)	1250 (47.7)	
**High cholesterol ratio**				
No	745 (21.1)	374 (41.2)	371 (14.2)	**< 0.0001**
Yes	2781 (78.9)	534 (58.8)	2247 (85.8)	

P values in bold are statistically significant. SD = standard deviation; HDL-C = high-density lipoprotein cholesterol; LDL-C = low-density lipoprotein cholesterol

### BMI and WC

For females, there is moderate agreement between BMI and WC in the group aged less than 40 years (Cohen’s kappa [k] = 0.54) and in the group aged 60 years and above (k = 0.42), while there is fair agreement in the group aged 40 to 59 years (k = 0.39; Table 3). For males, there is moderate agreement in the group aged less than 40 years (k = 0.50) and in the group aged 40 to 59 years (k = 0.48), while there is fair agreement for the group aged 60 years and above (k = 0.34; Table 3).

**Table 3:** Agreement between BMI, WC, WHR and NC by age groups and gender

	Females		Males	
Age group	Kappa (k) value	Interpretation	Kappa (k) value	Interpretation
**18–39, years**				
BMI-WC	0.54	Moderate	0.50	Moderate
BMI-WHR	0.22	Fair	0.12	Poor
BMI-NC	0.12	Poor	0.01	Poor
WC-WHR	0.51	Moderate	0.33	Fair
WC-NC	0.22	Fair	0.02	Poor
WHR-NC	0.07	Poor	0.00	Equal to chance
**40–59, years**				
BMI-WC	0.39	Fair	0.48	Moderate
BMI-WHR	0.07	Poor	0.12	Poor
BMI-NC	0.01	Poor	0.00	Equal to chance
WC-WHR	0.36	Fair	0.36	Fair
WC-NC	0.02	Poor	0.00	Equal to chance
WHR-NC	0.03	Poor	0.00	Equal to chance
**≥ 60, years**				
BMI-WC	0.42	Moderate	0.34	Fair
BMI-WHR	0.31	Fair	0.06	Poor
BMI-NC	0.23	Fair	0.02	Poor
WC-WHR	0.65	Moderate	0.23	Fair
WC-NC	0.09	Poor	0.00	Equal to chance
WHR-NC	0.12	Poor	0.12	Poor

BMI = body mass index; NC = neck circumference; WC = waist circumference; WHR = waist-hip ratio

Table 4 shows that there is moderate agreement between BMI and WC in all ethnicities for both males and females.

**Table 4:** Agreement between BMI, WC, WHR and NC by ethnicity and gender

	Females		Males	
Ethnic groups	Kappa (k) value	Interpretation	Kappa (k) value	Interpretation
**Local Emirati**				
BMI-WC	0.55	Moderate	0.52	Moderate
BMI-WHR	0.19	Poor	0.19	Poor
BMI-NC	0.34	Fair	0.09	Poor
WC-WHR	0.52	Moderate	0.42	Moderate
WC-NC	0.46	Moderate	0.12	Poor
WHR-NC	0.25	Fair	0.06	Poor
**Arab Non-local**				
BMI-WC	0.66	Moderate	0.55	Moderate
BMI-WHR	0.35	Fair	0.15	Poor
BMI-NC	0.00	Equal to chance	0.00	Equal to chance
WC-WHR	0.56	Moderate	0.32	Fair
WC-NC	0.00	Equal to chance	0.00	Equal to chance
WHR-NC	0.00	Equal to chance	0.00	Equal to chance
**Asian non-Arab**				
BMI-WC	0.41	Moderate	0.47	Moderate
BMI-WHR	0.18	Poor	0.13	Poor
BMI-NC	0.00	Equal to chance	0.00	Equal to chance
WC-WHR	0.46	Moderate	0.34	Fair
WC-NC	0.00	Equal to chance	0.00	Equal to chance
WHR-NC	0.00	Equal to chance	0.00	Equal to chance

BMI = body mass index; NC = neck circumference; WC = waist circumference; WHR = waist-hip ratio

### BMI and WHR

The analysis found fair-to-poor agreement between BMI and WHR for both males and females and for different age groups and ethnicities (k ranging from 0.06 to 0.31; Tables 3 and 4).

### BMI and NC

The agreement between BMI and NC was equal to chance (k = 0.0) or poor (k = 01.2–0.01) for all categories, except for females aged 60 years and above (fair; k = 0.23) and Emirati females (fair; k = 0.34). The agreement was fair for all male categories except for Emirati citizens, where it was moderate (k = 0.42; Tables 3 and 4).

### WC and WHR

The agreement between WC and WHR was moderate for all female categories (k = 0.65–0.46) except for females aged 40–49 years, where it was fair (k = 0.36; Tables 3 and 4).

The agreement for all male categories was fair (k = 0.32–0.36), except for Emirati citizens, where agreement was moderate (k = 0.42; Tables 3 and 4).

### WC and NC

The agreement between WC and NC ranged from poor to equal to chance for both males and females (k = 0.00–0.12; Tables 3 and 4).

### WHR and NC

The agreement between WHR and NC for both males and females was equal to chance for both males and females (k = 0.06) except for Emirati citizens and people aged 60 and above, where it was poor (k = 0.12; Tables 3 and 4).

### Association between overweight/obesity and cardiometabolic risks

Table 5 shows multivariate binary logistic analyses for anthropometric measurements as predictors for cardiometabolic risk factors after adjusting for age, gender, nationality, smoking status and physical activity. Overweight/obesity identified by WHR, WC and BMI are predictors of diabetes, with odds ratios [(ORs) (95% confidence interval (CI)) of 1.82 (1.45–2.28), 1.52 (1.20–1.92) and 1.49 (1.17–1.90)], respectively. Overweight/obesity identified by WC, WHR and BMI are predictors of hypertension, with ORs (95% CI) of 1.62 (1.35–1.96), 1.52 (1.27–1.83) and 1.44 (1.20–1.74), respectively. Overweight/obesity according to NC predicts neither diabetes nor hypertension. Overweight/obesity identified by BMI is a better predictor than other indices for dyslipidaemia, except for hypercholesterolaemia. The WHR index does not show significant prediction for hypercholesterolaemia and high low-density lipoprotein cholesterol (LDL–C; Table 5). Overall, BMI and WC showed better prediction of hypertension, diabetes and all types of dyslipidaemia followed by WHR, while NC did not show any associations with diabetes or hypertension, except for dyslipidaemia.

**Table 5:** Binary logistic analyses of association between overweight/obesity based on different anthropometric measurements and cardiometabolic risk factors, Odds Ratio (95% Confidence Interval)

Variable	BMI	WC	WHR	NC
**Diabetes Mellitus**				
No	1	1	1	1
Yes	1.49 (1.17–1.90)	1.52 (1.20–1.92)	1.82 (1.45–2.28)	0.82 (0.61–1.09)
P value	**0.001**	**0.001**	**< 0.0001**	0.169
**Hypertension**				
No	1	1	1	1
Yes	1.44 (1.20–1.74)	1.62 (1.35–1.96)	1.52 (1.27–1.83)	0.95 (0.73–1.24)
P value	**< 0.0001**	**< 0.0001**	**< 0.0001**	0.730
**Hypercholesterolemia ≥ 5.2 mmol/l**				
No	1	1	1	1
Yes	1.30 (1.10–1.53)	1.37 (1.16–1.61)	1.07 (0.91–1.26)	1.27 (1.01–1.60)
P value	**0.002**	**< 0.0001**	0.430	**0.045**
**Hypertriglyceridemia ≥ 1.7 mmol/l**				
No	1	1	1	1
Yes	1.89 (1.58–2.25)	1.57 (1.32–1.87)	1.38 (1.16–1.63)	1.34 (1.04–1.72)
P value	**< 0.0001**	**< 0.0001**	**< 0.0001**	**0.023**
**Low-HDL–C** **< 1 mmol/l for males &** **< 1.3 mmol/l for females**				
No	1	1	1	1
Yes	1.83 (1.54–2.17)	1.54 (1.30–1.82)	1.56 (1.32–1.84)	1.52 (1.20–1.92)
P value	**< 0.0001**	**< 0.0001**	**< 0.0001**	**0.001**
**High-LDL–C** **≥ 3.4 mmol/l**				
No	1	1	1	1
Yes	1.44 (1.22–1.70)	1.31 (1.11–1.54)	1.15 (0.98–1.36)	1.39 (1.10–1.76)
P value	**< 0.0001**	**0.001**	0.092	**0.007**
**High cholesterol ratio (TC/HDL) ≥ 3.4 mmol/l**				
No	1	1	1	1
Yes	2.65 (2.15–3.26)	2.38 (1.90–2.97)	1.84 (1.46–2.31)	1.96 (1.47–2.62)
P value	**< 0.0001**	**< 0.0001**	**< 0.0001**	**< 0.0001**

Adjusted for Sex, age (years), nationality, physical activity, and smoking

BMI = body mass index; NC = neck circumference; WC = waist circumference; WHR = waist-hip ratio

## Discussion

This study reveals the high burden of above-normal body weight in adult populations in the UAE based on various anthropometric measures. Among the measures examined, the data identified moderate agreement between BMI and WC, moderate-to-fair agreement between WC and WHR, and poor agreement between BMI and WHR, WC and NC, and WHR and NC. The study did not show significant differences in agreement based on gender, age group or ethnicity. However, Nevill and Metsios [18] called for redefining overweight and obesity cutoff values based on age groups and gender, as adiposity levels may vary according to these factors.

The study results are consistent with Haregu et al. [19] who had similar findings among a Kenyan population, with moderate agreement between BMI and WC, fair agreement between WC and WHR, and poor agreement between BMI and WHR. Similarly, a large study [20] that included participants from 29 countries in Asia, Europe, Latin America and North America reported good agreement between BMI and WC in high- and low-weight subjects and moderate agreement in subjects with intermediate weight. Moreover, Rona et al. [21] showed good agreement between BMI and WC among participants from Germany and United Kingdom; however, their study was conducted only among men in the military.

Agreement and correlation are commonly used to assess the relationship between two variables or measurements. Several studies have examined the relationship between different anthropometric measurements of obesity using the correlation statistical test [10, 22–24]. Correlation measures the degree of overall linear relationship between measurements but cannot tell whether two measurements can be used interchangeably [25]. Limited literature, including this study, have assessed the agreement between anthropometric measurements and indices of obesity using the concordance or agreement statistical test. This test measures the degree of concordance or agreement between measurements based on categorical classifications of weight status [25] and should be used to judge whether two obesity anthropometric measurements or indices were concordant and can be used interchangeably.

Several studies showed that obesity (BMI ≥ 30 kg/m^2^) has higher health risk than overweight (BMI = 25–29.9 kg/m^2^) [26]. However, both overweight and obesity were defined by the WHO as abnormal or excessive fat accumulation that may impair health and can be used as indicators for high health risk [27]. This study shows an alarming prevalence of overweight and obesity in the UAE requiring regular monitoring and screening. BMI is recognized worldwide as a screening measurement for overweight and obesity but can be challenging to calculate in community settings. Both WC and WHR can be difficult to measure in conservative populations, particularly in females. NC is easy to perform and can be a culturally acceptable measurement in the UAE community [9, 10, 28]. However, this study showed poor agreement between NC and other obesity anthropometric measurements and indices. Furthermore, the study did not show associations between NC and metabolic syndrome risk, diabetes or hypertension, except for dyslipidaemia. These findings are not consistent with several studies that demonstrated that NC is an accurate and reliable alternative for assessing overweight and obesity and predicting metabolic syndrome [9–11]. Furthermore, a systematic review and meta-analysis conducted by Moradi et al. [29] demonstrated that larger NC was associated with the risk of hypertension among Western ethnicities but not Eastern ethnicities. This inconsistency could be caused by inappropriate cutoff values used for NC or because anthropometric measures and indices are treated as categorical variables rather than continuous variables during agreement statistical analysis. To the best of our knowledge, no study has created unique reference cutoff values for NC in the UAE. Studies are needed to create reference neck circumference cutoff values for UAE adult populations in order to use it as a screening tool and for community-based research.

This study indicated that BMI, WC and WHR were associated with hypertension, diabetes and all types of dyslipidaemia, although WHR was not associated with high LDL–C. Similarly, Haregu et al. [16] reported WC and WHR as the strongest predictors of hypertension and hyperglycaemia, while BMI was a stronger predictor of hypercholesterolaemia. Moreover, the study results are consistent with several studies reporting that BMI, WC and WHR are strong predictors of metabolic syndrome [7, 12, 13].

From a public health perspective, our findings indicate that the burden of overweight and obesity in the UAE is alarming and poses great health challenges and concerns. It is associated with cardiovascular risks (diabetes, hypertension and dyslipidaemia). The Global Burden of Disease Study in 2017 reported that cardiometabolic diseases disease represents the leading cause of premature death in the UAE, causing approximately 40% of all deaths [30]. Therefore, prompt preventive actions should be implemented targeting the UAE population at a young age to lessen the risks of overweight and obesity. Increasing public awareness, promoting lifestyle modifications and introducing health policies that emphasise regular weight screening using convenient anthropometric measurement at primary health care centres, nurseries and schools are highly recommended. Moreover, weight loss with medications and bariatric surgery should be considered by physicians for individuals at high risk of obesity consequences.

This study has some limitations. First, the cross-sectional design of the research cannot establish temporality between anthropometric measurements and cardiometabolic risks. Second, the study was limited to adult populations in the UAE; thus, our results may not be generalised to young populations. Thirds, this study assessed statistically the agreement between anthropometric measurements based on dichotomous categorical classifications of weight status which might cause loss of precision of estimated mean of the continuous variables. Last, heterogeneity in cutoff values among different ethnic populations was not considered for all anthropometric measurements. There has been some debate around the world over whether these cutoff values for overweight and obesity should be applied universally. Different limits for defining overweight and obesity for populations in the Asia-Pacific area were recommended in a report cosponsored by the WHO Western Pacific Region [31].

## Conclusions

Overall, there was a moderate to a poor agreement between BMI, WC, WHR and NC. Particularly, NC showed poor agreement with BMI, WC and WHR and this may warrant further investigations. BMI and WC showed better performance for identifying cardiometabolic risks than WHR and NC.

## Data Availability

The datasets used and/or analysed during the current study are available from the corresponding author on reasonable request.

## Abbreviations

WHO: World Health Organisation
BMI: Body Mass Index
NC: Neck circumference
WC: Waist circumference
WHR: Waist hip ratio
